# Gold-Coated Iron Composite Nanospheres Targeted the Detection of *Escherichia coli*

**DOI:** 10.3390/ijms14036223

**Published:** 2013-03-18

**Authors:** Ugur Tamer, Demet Cetin, Zekiye Suludere, Ismail Hakkı Boyaci, Havva Tumay Temiz, Hande Yegenoglu, Philippe Daniel, İlker Dinçer, Yalçın Elerman

**Affiliations:** 1 Department of Analytical Chemistry, Faculty of Pharmacy, Gazi University, Etiler, Ankara 06330, Turkey; E-Mail: ednah87@hotmail.com; 2 Science Teaching Programme, Faculty of Education, Gazi University, Besevler, Ankara 06330, Turkey; E-Mail: demetcetin@gazi.edu.tr; 3 Department of Biology, Faculty of Science, Gazi University, Besevler, Ankara 06330, Turkey; E-Mail: zekiyes@gazi.edu.tr; 4 Department of Food Engineering, Hacettepe University, Beytepe, Ankara 06530, Turkey; Food Research Center, Hacettepe University, 06800 Beytepe, Ankara, Turkey; E-Mails: ihb@hacettepe.edu.tr (I.H.B.); tumaytemiz89@gmail.com (H.T.T.); 5 Institut des Molécules et Matériaux du Mans IMMM, LUNAM Université, Université du Maine, UMR CNRS 6283 F–72085 Le Mans 9, France; E-Mail: daniel@univ-lemans.fr; 6 Department of Engineering Physics, Faculty of Engineering, Ankara University, Besevler, Ankara 06100, Turkey; E-Mails:idincer@eng.ankara.edu.tr (İ.D.); elerman@ankara.edu.tr (Y.E.)

**Keywords:** magnetic gold nanoparticle, SERS, immunomagnetic separation, *E. coli*, surface functionalisation of particles

## Abstract

We report the preparation and characterization of spherical core-shell structured Fe_3_O_4_–Au magnetic nanoparticles, modified with two component self-assembled monolayers (SAMs) consisting of 3–mercaptophenylboronic acid (3–MBA) and 1–decanethiol (1–DT). The rapid and room temperature synthesis of magnetic nanoparticles was achieved using the hydroxylamine reduction of HAuCl_4_ on the surface of ethylenediaminetetraacetic acid (EDTA)-immobilized iron (magnetite Fe_3_O_4_) nanoparticles in the presence of an aqueous solution of hexadecyltrimetylammonium bromide (CTAB) as a dispersant. The reduction of gold on the surface of Fe_3_O_4_ nanoparticles exhibits a uniform, highly stable, and narrow particle size distribution of Fe_3_O_4_–Au nanoparticles with an average diameter of 9 ± 2 nm. The saturation magnetization value for the resulting nanoparticles was found to be 15 emu/g at 298 K. Subsequent surface modification with SAMs against glucoside moieties on the surface of bacteria provided effective magnetic separation. Comparison of the bacteria capturing efficiency, by means of different molecular recognition agents 3–MBA, 1–DT and the mixed monolayer of 3–MBA and 1–DT was presented. The best capturing efficiency of *E. coli* was achieved with the mixed monolayer of 3–MBA and 1–DT-modified nanoparticles. Molecular specificity and selectivity were also demonstrated by comparing the surface-enhanced Raman scattering (SERS) spectrum of *E. coli*-nanoparticle conjugates with bacterial growth media.

## 1. Introduction

Fabrication of magnetic nanoparticles and their composites has become more popular during last decade due to their potential applications in fields such as medical imaging, drug delivery, therapy, catalysis, and sensors [1–5]. In particular, core–shell structured magnetic nanoparticles attract more attention due to biomedical applications including the separation and purification of biomolecules from matrices [6–9]. Therefore, the synthesis of magnetic nanocomposites would be a crucial step for potential applications in the next decade [10]. The well-known surface chemistry of gold has been proven to provide the stability to the magnetic nanoparticles in solution, as well as helping with binding the various chemical and biological agents [11–16]. The most common approach involves a citrate or borohydride reduction of a gold salt to produce gold nanoparticles [11,12,17]. Gold-coated iron nanoparticles were formed inside the reverse micelle by the reduction of a metal salt using sodium borohydride [11,18,19]. Zhou *et al.*, prepared gold-coated iron nanoparticles using reverse micelles characterized by Transmision electron microscopy (TEM) [20]. The formation of a gold shell on the magnetic nanoparticle was also performed by an iterative reduction method using hydroxylamine as a reducing agent [21,22]. The optical and magnetic properties of the fluorescent silica-coated gold nanorods and magnetic nanorods were also reported [23,24]. However, the production of monodispersed, nanometer sized gold-coated magnetic particles remains as a significant challenge.

Immunomagnetic separation (IMS) has become a very popular approach and involves immobilizing a selective probe, such as antibodies or aptamers, to particles to directly trap target biomolecules from liquid media. In the study of El–Boubbou *et al.*, a magnetic glyco-nanoparticle (MGNP)-based system was demonstrated to not only detect *E. coli*, but also remove up to 88% of the target bacteria from the medium [25]. Cheng *et al.* functionalized magnetic nanoparticles by immobilizing an anti-*E. coli* antibody on the surface of amine-functionalized magnetic nanoparticles to fabricate biofunctional magnetic nanoparticles (BMNPs), which could concentrate traces of *E. coli* from sample solutions with high capture efficiency [26]. An MGNP-antibody conjugate was used to capture *E. coli* O157:H7 in beef samples and separate them using a magnet with high capture efficiency [27]. The use of magnetic nanoparticles has opened new dimensions in immunomagnetic separation. From a functionality point of view, nanoparticles are used as magnetic carriers or labels in a variety of applications. Magnetic nanoparticles are very useful for the separation of target cells from the mixture of bacteria and food matrices and also help to concentrate separated cells into a very small volume.

The use of magnetic nanoparticles also has advantages in assay sensitivity, speed, and precision. All of the bioseparation and medicinal applications require that these nanoparticles have high magnetization and a small diameter with narrow particle size distribution. In the study of Gupta and Curtis, superparamagnetic nanoparticles with a specific shape and size were prepared and coupled to various proteins. The silica-coated superparamagnetic nanoparticles with the uniform diameter of approximately 60 nm were synthesized and the magnetic nanoparticles were modified with *N*–(2–aminoethyl)–3–aminopropyltrimethoxysilane. The immunomagnetic nanoparticles were then successfully prepared by covalently immobilizing anti–CD34+ monoclonal antibodies to the surface of amino silane-modified magnetic particles. The cell separation results showed that the synthesized immunomagnetic nanoparticles could rapidly and conveniently separate the CD34+ cells with high efficiency and specificity compared to normal ones [28,29].

Micro and nano-structured surface-enhanced Raman scattering (SERS) substrates have enabled the detection of pathogens present in biofluids [30]. Several publications have focused on determining the spectral bands that are characteristic of bacteria from different species and cell lines [31,32]. In many instances, the spectra of commonly used bacterial growth media are similar to published spectra purportedly characteristic of specific bacterial species [33]. The differences observed in the SERS reports are mainly due to different interactions of the SERS–active media with different cell components. It is however possible to limit these differences, for instance with reproducible nanoparticle production and also with appropriate surface modification. Self-assembled monolayers (SAMs) offer a number of advantages as platforms for suitable surface modification to attach biological molecules onto nanoparticle surfaces. SAMs of Raman reporter molecule (5,5–dithiobis (2–nitrobenzoic acid), DTNB), were coated on the surface of the rod-shaped gold nanoparticles to enumerate *Escherichia coli* in the method combining IMS and SERS [34]. The formation of SAMs from dilute ethanolic solution is straightforward experimentally, and applicable to most sensor configurations. SAMs form structurally well-defined and compact monolayers on the surfaces of the nanoparticles. In addition, these systems have been studied extensively, and their chemistry is well-understood. Recently, 3–aminophenylboronic acid (APBA) was immobilized on a gold electrode via a self-assembled monolayer and the change in capacitance of the sensing surface caused by the binding between 3–APBA and bacteria in a flow system was detected by a potentiostatic step method [35]. Since the method is not specific to the type of bacteria, it shows the binding of bacteria to the 3–APBA. The complexation of saccharides with aromatic boronic acids produces a stable ester where the binding constant is dependent on the pH, electrolyte concentration and pKa of the aromatic boronic acid [36]. Since a bacterium cell will consist of polysaccharides with diol-groups that can chemoselectively bind to 3–APBA, it would be useful to apply 3–APBA to a rapid detection of total bacteria [37,38]. Recent work by Van Duyne’s group also demonstrated that glucose was partitioned into an alkanethiolate monolayer, such as decanethiol [39]. The alkyl spacer functional group was used to increase glucose permeation for the direct detection of glucose using SERS.

Specifically, the method of preparation of the SERS-active substrate and the way in which it is brought in contact with the bacteria have strong effects on the spectra in which differences can be attributed to the above characteristics. The main objective of this study is to develop a simple and effective SERS substrate, which provides a well-defined recognition surface containing the alkyl spacer and phenylboronic acid functional group for antibody-free bacteria detection. The rapid and room temperature reaction synthesis of core–shell Fe_3_O_4_–Au structured monodispersed magnetic nanoparticles was achieved using the hydroxylamine reduction of HAuCl_4_ onto ethylenediaminetetraacetic acid (EDTA)-immobilized iron nanoparticles in the presence of an aqueous solution of hexadecyltrimetylammonium bromide (CTAB). The resulting nanoparticles were characterized by TEM, ultraviolet visible spectroscopy (UV–Vis), and X-Ray Diffraction (XRD), and the magnetic properties of the nanoparticles were also examined. Gold-coated iron nanoparticles were modified with two-component self-assembled monolayers consisting of 3–MBA and 1–DT. *E. coli* was chosen as a model system and magnetic nanoparticles with different functional groups were compared with respect to the capturing efficiency of *E. coli*. After the interaction of nanoparticle-*E. coli*, scanning electron microscopy (SEM) images were taken. The potential suitability of the developed substrate as a SERS platform which is able to recognize *E. coli* by directly attaching to the magnetic surface was also assessed.

## 2. Results and Discussion

### 2.1. Characterization of Core Shell Structured Magnetic Nanoparticles

To date, several methods have been developed based on wet chemistry approach to synthesize magnetic gold nanoparticles with controllable size and aspect ratios [24]. In the present work, a different strategy was employed to construct a core-shell structured Au–Fe_3_O_4_ hybrid particle which exhibited the optical properties of Au metal and the magnetic properties of Fe_3_O_4_. This method involved a two-steps process. Firstly, pre-prepared Fe_3_O_4_ nanoparticles were homogeneously dispersed in an EDTA solution. Afterwards, in the presence of the mixture of surfactant solution CTAB and gold chloride salt, the hydroxylamine solution was used to reduce Au^3+^ into Au° and gold was reactively deposited onto the EDTA-immobilized Fe_3_O_4_ particles. In order to stabilize and obtain smoother and homogenous gold layer, gold precursor ions were directly reduced by hydroxylamine. The gold coating process was carried out in the presence of CTAB and EDTA, to give better particle monodispersity and avoid agglomeration problems associated with ionic interactions. The corresponding polydispersity index (PDI) of gold coated iron nanoparticles was 0.24. After coating a gold layer onto the iron nanoparticle, the zeta potential increased from −47 mV to +15 mV. Nine months after the preparation of the gold-coated iron nanoparticle, coagulation or agglomeration was not observed due to the presence of a CTAB layer on the magnetic particle surface.

**Figure 1:**
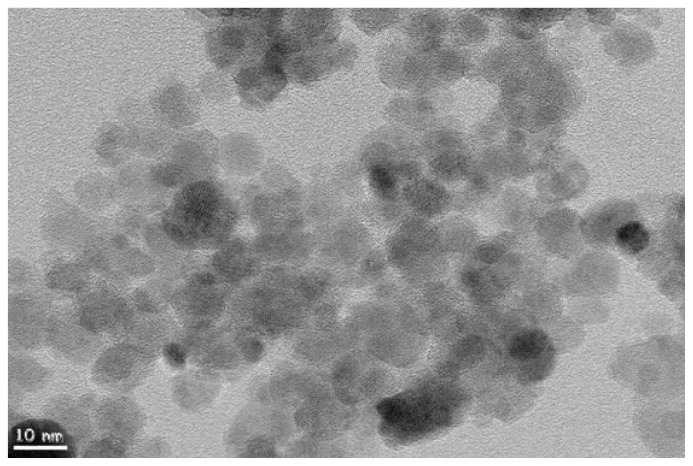
Transmision electron microscopy (TEM) image of magnetic gold nanoparticles.

Figure 1 shows representative TEM image of spherical-shaped gold iron nanoparticles. It was found that large quantities of well-defined nanoparticles were obtained and the products consisted of large amount of monodisperse nanoparticles. The shape distributions were obtained by measuring individual particles from TEM images and the spherical-shaped gold coated iron nanoparticles had a diameter of 9 ± 2 nm.

Nanoparticles spherical shape can be clearly seen from atomic force microscopy (AFM) measurements (Figure S1a). According to AFM measurements, grain analysis was done with the threshold value of 6 nm and 685 grains count for these nanoparticles. The radius values of individual nanoparticles were used for nanoparticles radius histogram shown in Figure S1b. Average Radius values of nanoparticles were found 23.5 nm by using image processing software and histogram analysis. The width of nanoparticles obtained from AFM analysis was found to be bigger than obtained data from TEM and XRD analysis. This contradiction arises from tip’s convolution effect. The percentage of spherical-shaped nanoparticles was close to 100%.

Figure S2 shows the extinction spectra of the spherical-shaped gold coated iron nanoparticles synthesized by using the EDTA-immobilized growth method. As shown in Figure S2, the spectrum for the spherical gold coated iron nanoparticle was readily distinguishable as compared to EDTA-immobilized Fe_3_O_4_ nanoparticles, and spherical gold-coated iron nanoparticles had a plasmon band at 545 nm.

**Figure 2:**
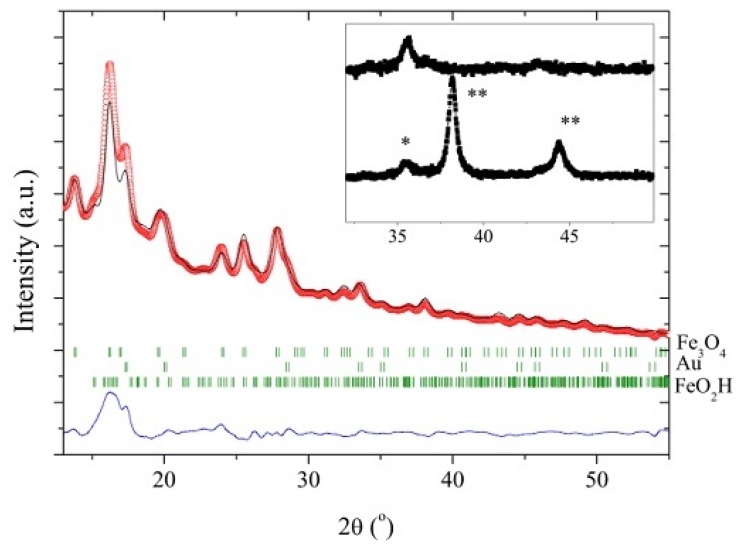
Rietveld refinement results of gold coated magnetic nanoparticles. Inset shows the X-Ray Diffraction (XRD) patterns with Cu Kα radiation for pure magnetic nanoparticles (top) and gold-coated magnetic nanoparticles (bottom). * shows the diffraction peaks of Fe_3_O_4_; ** shows the diffraction peaks of gold.

The gold-coated magnetic nanoparticles were also characterized using an XRD method. Figure 2 shows the Rietveld refinement results of XRD patterns for the gold-coated magnetic nanoparticles. According to the Rietveld refinement results, the unit cell parameters of Fe_3_O_4_ and gold were 8.359 (2) Å and 4.088 (2) Å for Fe_3_O_4_ and Au, respectively. The Bragg peaks of FeO_2_H were observed in the XRD diffraction pattern. The XRD analysis showed that the proportions were 57% Fe_3_O_4_, 5% Gold and 38% FeO_2_H. The inset in Figure 2 shows the X-ray powder diffraction pattern with Cu Kα radiation of pure magnetic nanoparticles and gold-coated magnetic nanoparticles, respectively. As labelled in Figure 2b, the peaks at 38.14°, 44.36° were assigned to the Au–Fe peaks of (111) and (200) respectively, which are located in the positions of the corresponding materials (JCPDS card No: 04–0784). The crystalline structure of gold iron nanoparticles has proven that the particles are face centred cubic with the dominant crystal planes of 111.

**Figure 3:**
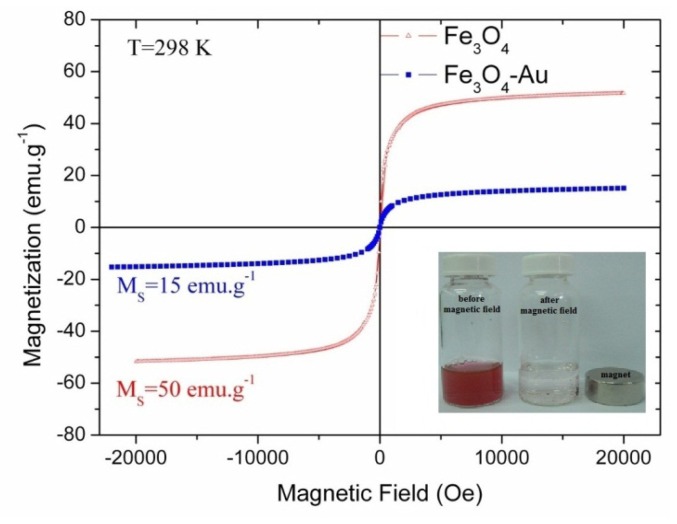
Magnetic field dependence of magnetic particles and inset: Photographs of magnetic gold nanosphere before and after applying magnetic field.

**Figure 4:**
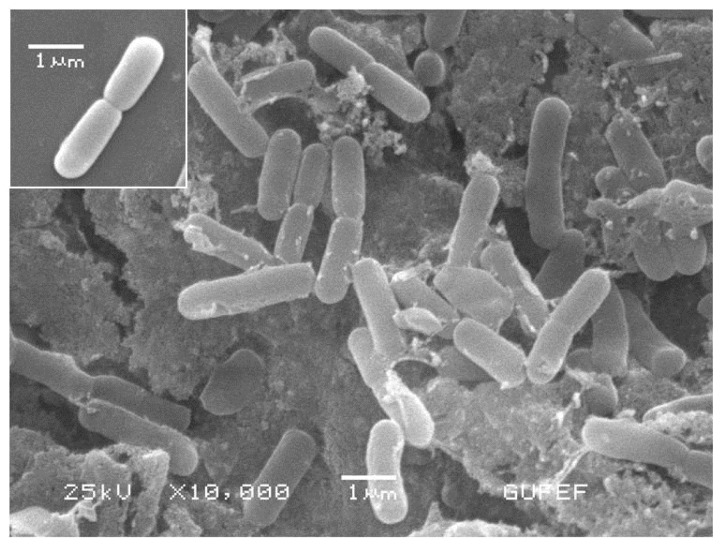
The scanning electron microscopy (SEM) images of *E. coli* immobilized with 3-MBA/1-DT modified magnetic nanoparticles on *E. coli*. Inset: The SEM image of unmodified *E. coli*.

The average crystalline size of the Fe_3_O_4_ core particles from the XRD pattern with Mo source was calculated by the Scherrer formula using the half-maximum full width of the Fe_3_O_4_ (311) peak. For this calculation, the diffractometer contribution to peak size was taken into account. It was found to be 6.1 nm, which is slightly smaller than, but in close agreement with the average particle size of nine nm for this sample derived from the TEM images. The magnetic measurements of the dried samples of the powders were performed at 298 K. The hysteresis loops measured for the uncoated and gold coated Fe_3_O_4_ nanoparticles are shown in Figure 3. As seen in the figure, the typical characteristics of superparamagnetic behavior are observed. The saturation magnetisation values from the magnetization curve in Figure 4 for the uncoated and gold-coated Fe_3_O_4_ nanoparticles were found to be 50 emu/g and 15 emu/g, respectively, at 298 K. The decrease of saturated magnetization was attributed to the formation of the gold layer. Magnetic gold nanoparticles synthesized by using the current system show strong magnetism, as indicated in the inset of Figure 3, and the effective separation of nanoparticles was achieved using only a magnet.

### 2.2. Interaction of Developed Core Shell Nanoparticles with *E. coli*

**Scheme 1:**
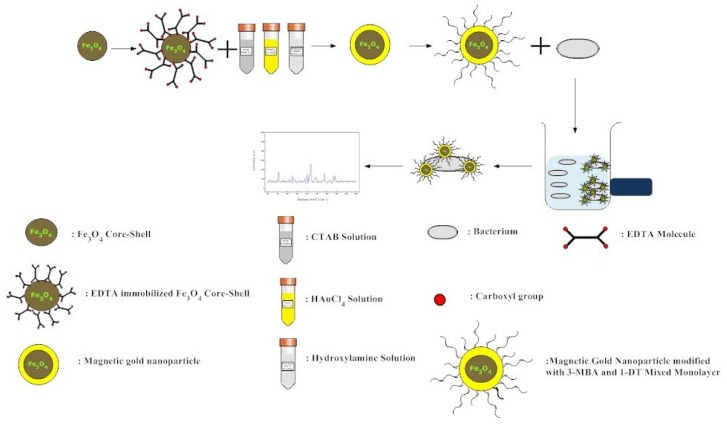
Schematic illustration of the surface modification of magnetic gold nanoparticles and *E. coli* assay.

A variety of methods for bacterial immobilization are based on antibody–antigen interactions, electrostatic and the physical entrapment of bacteria [40]. The physical entrapment and electrostatic methods suffer from low loading. Antibody–antigen interactions take advantage of the specific interaction. However, antibody-modified particles have been used in IMS and resulted in low immobilization efficiency [41]. Other non-pathogenic microorganisms including yeasts such as Saccharomyces cerevisiae have also been shown to interact with alkanethiolate groups of other SAM surfaces [28]. The choice of suitable functional group could affect the performance of IMS due to the support analyte interactions and surface reactivity. The performance of the magnetic gold nanoparticle is tested by using the magnetic separation of E. coli and SERS as a benchmark system for E. coli detection. In the present study, E. coli was selected as target due to the presence of glucoside moieties on the bacteria membrane [6]. For this purpose, the Fe_3_O_4_–Au nanoparticles can be fully dispersed in PBS solution and the magnetic properties and utility of gold iron nanoparticles were performed by the magnetic separation of the attached functional group as the mixture of 3–MBA and 1–DT on the gold shell. The schematic illustration for the formation of gold iron nanoparticle and subsequent attachment of SAMs for the binding of E. coli is shown in Scheme 1. The alkyl and phenylboronic acid functional group was used to increase the E. coli permeation for the direct SERS detection.

To verify and confirm the SAMs, bacteria binding reactivity and the magnetic separation capability, a colony counting method was used. In this experiment, resulting modified gold iron nanoparticles were coupled to the *E. coli* via aforementioned functional groups forming *E. coli*-immobilized Fe_3_O_4_–gold nanoparticles. Magnetic nanoparticles with functional groups were compared with respect to the capturing efficiency of *E. coli*. A magnetic field was applied to collect the magnetically active bacteria. Colonies of *E. coli* were counted to determine the percentage of the captured *E. coli*. Capturing efficiencies of the mixed monolayer of 3–MBA and DT, 3–MBA monolayer, DT monolayer and CTAB double layer were found to be 71%, 16%, 47% and 33%, respectively. The results for the 3–MBA monolayer- and ODT monolayer-modified nanoparticles were found to be inconsistent within parallel plates. The IMS percentage for CTAB modified nanoparticles in separation medium from bacterial growth solution became sufficient; however, the best capturing efficiency of *E. coli* was achieved with the mixed monolayer of 3–MBA and 1–DT. In our previus paper, we have developed a new magnetic approach to immunoassay detection of *E. coli*[9]. The antibody immobilized magnetic nanoparticles were reacted with *E. Coli* and the magnetic separation of these nanoparticles was easily accomplished only by a magnet. IMS percentages were varied in a range of 52.1%–21.9% and were dependent on the initial bacteria counts. The immunological based techniques, where antibodies were used as recognition agents providing high sensitivity and selectivity. However, antibodies also have some limitations as recognition elements in bioassays and biosensors. First, antibody production is quite expensive and time-consuming. Also, utilizing animals in antibody production causes some ethical problems. Antibodies are functional under specific environmental conditions which can be unfavorable for the analysis of environment or food samples. These limitations of antibodies led to the development of alternative recognition molecules [42]. The formation of molecular probes around nanoparticles involves a one-step process and it interacts preferably with the analyte. The enhanced number of functional groups by using SAMs on nanoparticle surface might increase the selectivity of different target molecules.

The specific reactivity between 3–MBA/1–DT and *E. coli* is also evidenced by SEM image, as shown in Figure 4. As a comparison, the SEM image of unmodified *E. coli* was also shown inset of Figure 4. It is likely that *E. coli* interacts with the functional groups on the surface of nanoparticles due to possible interactions between the bacterial membrane and SAMs of magnetic nanoparticles. The effective binding of 3–MBA/1–DT to *E. coli* could be based on the presence of an alkanethiolate group that acts as a partition layer within the outer phospholipid membrane layer of bacteria. On the other hand, phenyl boronic acid group (as a complexing agent on magnetic nanoparticle surface) that interacts with the carbohydrate groups of outer membrane lipopolysaccharides.

**Figure 5:**
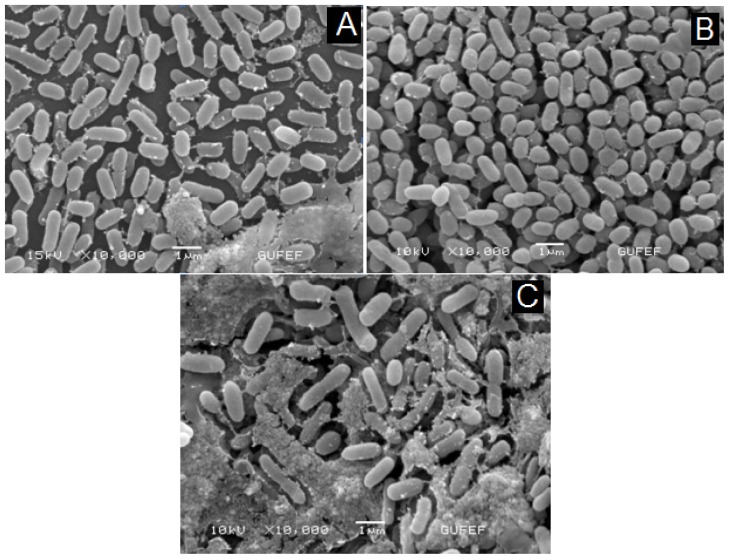
The SEM images of *E. coli* immobilized with (**A**) 3–MBA; (**B**) 1–DT; (**C**) CTAB–modified magnetic nanoparticles.

To demonstrate the immobilization of core–shell structured magnetic nanoparticles with different functional groups and *E. coli* interactions, SEM measurements were also carried out. The magnetic separation of these nanoparticles was easily accomplished and magnetic gold nanoparticles were separated only by a magnet. All of the SAMs, 3–MBA, 1–DT and CTAB-modified magnetic gold nanoparticles were coupled to the *E. coli*, forming *E. coli*-immobilized nanoparticles for capturing bacteria. The SEM images of *E. coli*-immobilized 3–MBA, 1–DT and CTAB-modified magnetic nanoparticles were indicated in Figure 5A–C, respectively. The results indicated that all of the SAMs listed above can, in principle, be targeted to achieve some degree of immobilization efficiency.

**Figure 6:**
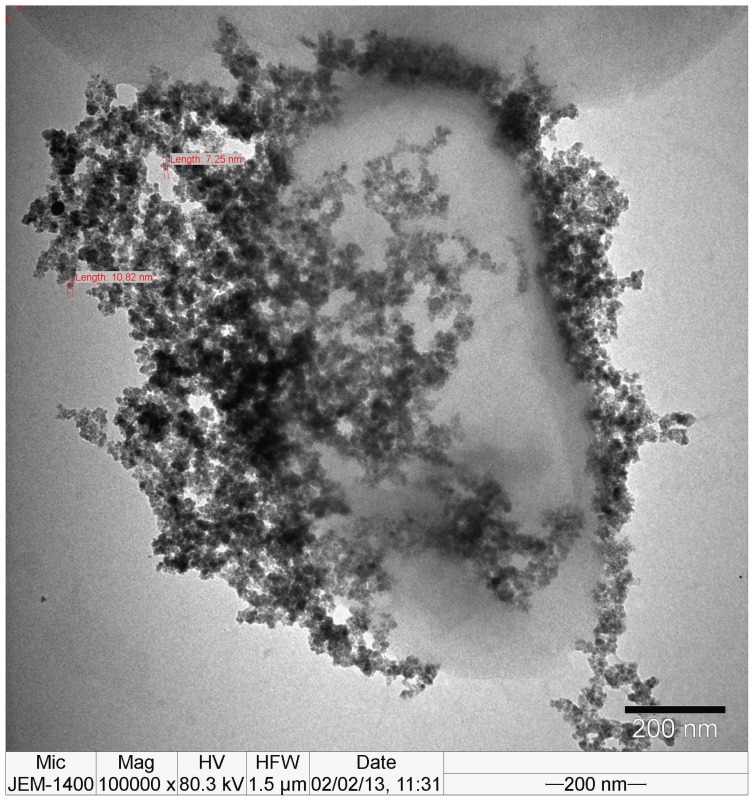
TEM image of *E. coli* immobilized with 3-MBA/1-DT modified magnetic nanoparticles.

We also carried out TEM experiments to demonstrate the immobilization of the magnetic nanoparticles on bacteria at high amplification. From TEM image as shown in Figure 6, higher amplification gave straightforward evidence to the immobilization of nanoparticles on the surface of *E. coli*.

**Figure 7:**
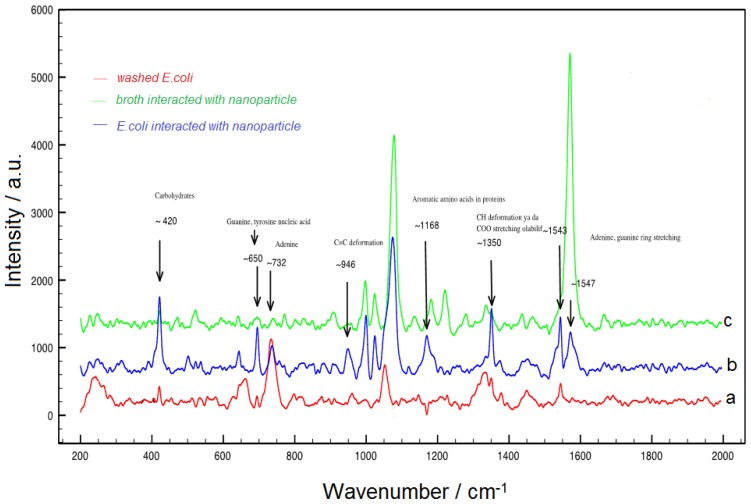
Surface-enhanced Raman scattering (SERS) spectrum of (**a**) 10^7^ cfu mL^−1^*E. coli*; (**b**) *E. coli* interacted with 3-MBA/1-DT-modified magnetic nanoparticles; (**c**) bacterial growth solution interacted with 3-MBA/1-DT-modified magnetic nanoparticles.

The molecular specificity of the SERS technique was studied by comparing the SERS spectrum of *E. coli*–nanoparticle conjugates with bacterial growth media. The SERS spectra for *E. coli* assays conducted by using SERS from magnetic gold nanoparticles are shown in Figure 7, which displays a typical SERS response after the addition of a concentration of *E. coli* (3.5 × 10^7^ cfu mL^−1^). The spectra contain features which are attributable to SERS of *E. coli* and are dominated by bands that are representative of the bacteria membrane protein. The SERS spectrum of bacterial growth media is different from the spectrum of *E. coli* membranes as shown in Figure 7. The effective separation of *E. coli* from the growth media was achieved by only a magnet, and as magnetic separation presents a time-saving replacement this allows the flexibility of applying the SERS detection method.

The identification of bacterial samples by SERS have also been conducted by mixing suspension of bacteria with suspension of 3-MBA/1-DT, 3–MBA, 1–DT, CTAB–modified colloidal magnetic nanoparticles as shown in Figure S3–S6. After interaction between nanoparticles and *E. coli*, the resulting SERS spectra were shown the characteristic bands of nanoparticle probe and bacteria.

## 3. Experimental Section

### 3.1. General

FeSO_4_7H_2_O, sodium hydroxide, hydrogen peroxide, sulphuric acid, and Tween 20 were purchased from Merck (Darmstadt, Germany). Hydrogen tetrachloroaurate (III) hydrate, FeCl_3_, 11–mercaptoundecanoic acid (11–MUA), 3–mercaptopropionic acid (3–MPA), hexadecyltrimetyl–ammonium bromide (CTAB), 98% ethanolamine, 3–MBA acid, 1–DT, ethylenediaminetetraacetic acid (EDTA), hydroxylamine hydrochloride, 2–morpholinoethanesulphonic acid monohydrate (MES), and absolute ethanol were obtained from Sigma–Aldrich (Taufkirchen, Germany). NaCl, Na_2_HPO_4_, and KH_2_PO_4_ were obtained from J.T. Baker (Deventer, The Netherlands), and were used as phosphate buffered saline (PBS). All reagents were used as received.

### 3.2. Preparation of Buffer Solutions

Phosphate buffered saline (PBS) (67 mM, pH 7.4) was prepared by using Na_2_HPO_4_, KH_2_PO_4_, and NaCl and the pH was adjusted with HCl or NaOH). PBST solution was prepared by mixing 67 mM, PBS, pH 7.4 with 0.05% Tween 20 (*v*/*v*). MES buffer (0.05 M, pH 6.5) was prepared by adjusting the pH with NaOH. All solutions were prepared with Milli–Q quality water (18 MΩ.cm) to reach the desired concentrations. Aqua regia solution (1:3 nitric acid/hydrochloric acids) was used to clean the quartz cuvettes and the glassware that was used to synthesize the nanoparticles.

### 3.3. Microorganisms

*E. coli* was obtained from Refik Saydam National Type Culture Collections, Ankara, Turkey. For *E. coli* detection, Luria Broth (LB) agar and LB broth was obtained from Laboratorios Conda (Madrid, Spain). The stock cultures for assay were grown on LB Broth at 37 °C for 18 h. The cultures were serially diluted (10-fold steps) with PBS and 100 μL diluted solutions of the cultures were plated. After incubation at 37 °C for 18 h, colonies on plates were counted to determine the number of colony forming units per milliliter (cfu mL^−1^).

### 3.4. Synthesis of Iron Nanoparticles

The Fe_3_O_4_ nanoparticles were prepared by co-precipitation of Fe (II) and Fe (III). The Fe(II)/Fe(III) ratio was kept at 0.5 in an alkaline solution. Briefly, 1.28 M FeCl_3_ and 0.64 M FeSO_4_·7H_2_O were dissolved in deionized water. The solution was then stirred vigorously until the iron salts were dissolved. Subsequently, a 100 mL solution of 1 M NaOH was added drop-wise into the mixture whilst stirring for 40 min. Precipitated magnetite is black in color. The black precipitate was collected on a permanent magnet and washed with deionized water. To obtain oxidized Fe_3_O_4_ nanoparticles, the resulting salt precipitates were first washed in 2 M HClO_4_ for three hours to oxidize iron salts and the color of the particle became brown under an argon atmosphere. The particles were then centrifuged for 20 min at 10,000 rpm. Subsequently, the supernatant solution was discarded and washed with deionized water. The washing procedure was performed three times.

### 3.5. Synthesis of Gold-Coated Fe_3_O_4_ Nanoparticles

The Au shell coating procedure was carried out in the presence of CTAB in order to encapsulate the iron nanoparticles with gold shells. Briefly, 10 mg Fe_3_O_4_ nanoparticles were suspended in 5 mL water before 0.27 M EDTA prepared in 1 M NaOH was added. The resulting solution was stirred in a sonicator for five minutes. The particles were then centrifuged for 10 min at 10,000 rpm. Subsequently, the supernatant was discarded and the particles washed with deionized water. The washing procedure was performed three times. The resulting precipitate was mixed with 7 mL 0.1 M CTAB, 3 mL 0.01 M HAuCl_4_ and 300 μL 1 M NaOH. The solution was then stirred vigorously and, subsequently, 150 mg hydroxylamine was added to the mixture and stirred vigorously for three minutes. The resulting nanoparticle solution color was dark red. The solution was stored at room temperature for 24 hours before use.

### 3.6. Surface Modification of Core–Shell Structured Magnetic Nanoparticles

To fabricate the capture probe for *E. coli*, magnetic gold nanoparticles were modified with different functional groups, such as 1–DT, 3–MBA and the mixture of 1–DT and 3–MBA in absolute ethanol overnight to form an SAM monolayer. Briefly, 5 mg gold–iron nanoparticles were transferred into the ethanol solution and centrifuged for 10 min at 10,000 rpm to remove CTAB from the particle surfaces. Then, the mixture of 1–DT and 3–MBA (1:1 molar ratio) was transferred into ethanol and magnetic gold nanoparticles were added, before the final mixture was left to stand for four hours in order to allow the thiol end groups to be chemisorbed onto the gold iron nanoparticles. The unreacted excess of 1–DT and 3–MBA was removed from the thiol-modified nanoparticles by applying a permanent magnet for 30 min followed by decantation of the supernatants and resuspension in ethanol. This procedure was repeated a minimum of three times. Nanoparticles were collected using a magnet and washed with 0.05 M MES (pH 6.5) buffer. In order to avoid non-specific interaction, 1% (*v*/*v*) ethanolamine was used to block active groups or any unblocking binding sites on the surface of magnetic gold nanoparticles. To prevent light-induced flocculation of the colloids and oxidation of the alkanethiolates, all nanoparticle solutions were stored in the dark and refrigerated at 4 °C. The same procedure was applied to form the 1–DT terminal groups.

### 3.7. Preparation of Nanoparticle–Bacteria Conjugates for the Determination of Capturing Efficiency

Bacteria (1 mL of *E. coli* (10^7^ cfu mL^−1^)) were washed twice with sterile PBS to remove the LB medium. One mililiter of each nanoparticle solution was washed twice with sterile PBS to remove ethanol, and the sterile PBS was discarded after washing steps. One mililiter of *E. coli* (10^7^ cfu mL^−1^) and nanoparticles were incubated for 30 min at 37 °C on an orbital shaker at 100 rpm. After the incubation period, nanoparticles were collected using a permanent magnet. In order to determine the number of unconjugated bacteria left in the buffer, 100 μL was taken from the remaining PBS and plated onto LB agar in triplicate. To remove the unconjugated and loosely conjugated bacteria, the nanoparticle-bacteria conjugate was then washed three times with sterile PBS in an ultrasound bath containing ultrapure water for 10 s. The washing volumes were collected separately and 100 μL was plated on LB agar. This procedure was performed for all SAM-modified nanoparticle solutions.

The capturing efficiency is described as the difference between the number of initial bacteria and the number of unbound and loosely bound bacteria divided by the initial number of bacteria multiplied by a hunderd. The number of unbound bacteria was determined using plate counting method as described above. It is also given as the formula below, where N_ib_ is number of initial bacteria and N_ub_ is number of unbounded bacteria:

$$\text{Capturing Efficiency}=(N_{\text{ib}}-N_{\text{ub}})/N_{\text{ib}}\times 100\%$$

### 3.8. Preparation of Nanoparticle-Bacteria Conjugates for SERS, SEM and TEM Measurements

The usability of the developed core–shell magnetic nanoparticles was investigated by magnetic separation of *E. coli* and SERS measurements. After the surface modification, magnetic gold nanoparticles were interacted w*ith E. coli* in bacterial growth solution for 30 min. Briefly, the modified magnetic gold nanoparticles (10 μL of 1 mg/mL) were mixed with 250 μL of *E. coli* (10^7^ cfu mL^−1^) and incubated for 30 min at 37 °C on an orbital shaker at 100 rpm. The nanoparticles were then separated magnetically and washed with sterile deionized water. After washing three times with sterile deionized water, the nanoparticle-bacteria conjugates were then resuspended in 250 μL sterile deionized water, and 10 μL of suspension was placed on the gold chip surface for SERS measurements. For control experiments, magnetic gold nanoparticles were interacted with *E. coli*-free bacterial growth medium. After interactions, magnetic nanoparticles were collected with a magnet and washed with deionized water.

For SEM images, samples of nanoparticle-bacteria conjugates were placed on small pieces of cover slips. They were then fixed overnight in 4% glutaraldehyde buffered with 0.2 M sodium phosphate (pH 7), and washed three times with 0.2 M sodium phosphate (pH 7) buffer. This was followed by dehydration in a graded series of ethanol (70, 80, 90, and 100%). Cells were then added to amyl acetate. The sample was next critical-point dried with CO_2_ (Polaron, CPD 7501) and coated with gold (Polaron SC 502 sputter coater). For TEM image, a small drop of the sample of nanoparticle-bacteria conjugates is deposited on the formvar-carbon coated grid, washed with deionized water, negatively stained with 2% uranyl acetate and examined by transmission electron microscope at 80 KV.

### 3.9. Instrumentation

Optical absorption spectroscopy measurements were performed in a Spectronics, Genesis model single beam spectrophotometer using 1 cm path length quartz cuvettes. Spectra were collected within a range of 300–800 nm. Transmission electron microscopy (TEM) samples were prepared by pipetting 10 μL of nanoparticle solution onto Formvar-Carbon coated copper grids and allowing the solution to stand for 10 min. TEM measurements were performed with the Tecnai G2 F30 instrument at 120 kV. TEM image of *E. coli* nanoparticle conjugate was obtained with JEOL JEM 1400 instrument at 80 kV X-Ray diffraction measurements were performed using of Xcalibur Gemini diffractometer equipped with an Eos CCD detector (Mo Kα radiation at a wavelength of 0.70903 Å) and Philips PW–1140 model diffractometer (Cu Kα radiation at a wavelength of 1.5406 Å).

DeltaNu Examiner Raman microscope (Deltanu Inc., Laramie, WY, USA) with a 785 nm laser source, a motorized microscope stage sample holder, and a CCD detector was used to detect *E. coli*. Instrument parameters were as follows: x20 magnification objective, 30 μm laser spot size, 100 mW laser power, and 60 s acquisition time. The average value of three SERS spectra was obtained and baseline correction was performed for all measurements. The magnetic field dependence of magnetization was measured by using a Vibrating Sample Magnetometer (VSM) from Microsense EV–9. Scanning electron micrographs were taken with JEOL JSM 6060 at 10–25 kV.

AFM characterizations of nanoparticles were performed by using a NT-MDT Pro Solver M atomic force microscope with semi-contact mode. AFM images were taken with Super Sharp, Diamond like Carbon (NSG\DLC 01) tips with curvature radius of 1–3 nm. Nanoparticles were scanned 2.08 Hz with resolution of 256 lines per image. For AFM measurements, nanoparticles were diluted and ultrasonicated in the Ortho-dichlorobenzene (ODCB) for 15 min and 10 μL of this dilution was dropped on to gold-coated mica substrate.

## 4. Conclusions

In this study, the immobilization efficiencies of SAMs consisting of 3–MBA, 1–DT, the mixed monolayer of 3–MBA and 1–DT and CTAB were evaluated. Suitable surface attachment chemistries provided the direct immobilization of biological recognition groups onto the surface of nanoparticles. This group successfully attached molecular recognition groups onto magnetic nanoparticle surfaces with immobilization procedures that were based on the use of multi-component self-assembled monolayers on nanoparticles as platforms for surface modification. Nevertheless, the choice of functional groups applied is a crucial factor affecting the stability of magnetic nanoparticles. CTAB-modified nanoparticles exhibited higher stabilities compared to MBA- and DT-modified nanoparticles. The fabrication of high quality hybrid core–shell nanoparticles endowed with sensing ability using optical and magnetic properties could show a novel SERS platform. The present work also demonstrated the effective magnetic separation for bacteria detection based on an alkanethiolate group that acts as a partition layer and phenyl boronic acid group as a complexing agent on a magnetic nanoparticle surface for capturing bacteria. The results demonstrated a molecular recognition agent mixed monolayer of SAM consisting of 3–MBA and 1–DT. For bacterial immobilization, surface polysaccharides could be targeted that are tightly attached to the bacterial cell wall. Direct attachment of biomolecules to functionalized nanoparticles is the preferred method of immobilization because of the increased stability. In addition, the presence of an organic thin film between the metal substrate and the biomolecule tends to minimize the problem of denaturation. Furthermore, non-specific adsorption can be minimized by the appropriate choice of SAM composition. Although there are several works published on related developments of SERS-based bacteria detection, the functional properties of the magnetic nanoparticles could give us an opportunity to perform the IMS and SERS detection.

## Supplementary Information



## References

[1] Brown K.R., Walter D.G., Natan M.J. (2000). Seeding of colloidal au nanoparticle solutions. 2. improved control of particle size and shape. Chem. Mat.

[2] Cheng F.Y., Su C.H., Yang Y.S., Yeh C.S., Tsai C.Y., Wu C.L., Wu M.T., Shieh D.B. (2005). Characterization of aqueous dispersions of Fe_3_O_4_ nanoparticles and their biomedical applications. Biomaterials.

[3] Cheng Y.X., Liu Y.J., Huang J.J., Li K., Zhang W., Xian Y.Z., Jin L.T. (2009). Combining biofunctional magnetic nanoparticles and atp bioluminescence for rapid detection of escherichia coli. Talanta.

[4] Wang S.F., Tan Y.M., Zhao D.M., Liu G.D. (2008). Amperometric tyrosinase biosensor based on Fe_3_O_4_ nanoparticles-chitosan nanocomposite. Biosens. Bioelectron.

[5] Zhang W., Yang T., Li X., Wang D.B., Jiao K. (2009). Conductive architecture of Fe_2_O_3_ microspheres/self-doped polyaniline nanofibers on carbon ionic liquid electrode for impedance sensing of DNA hybridization. Biosens. Bioelectron.

[6] Xu H., Cui L.L., Tong N.H., Gu H.C. (2006). Development of high magnetization Fe_3_O_4_/polystyrene/ silica nanospheres via combined miniemulsion/emulsion polymerization. J. Am. Chem. Soc.

[7] Meldrum F.C., Heywood B.R., Mann S. (1992). Magnetoferritin-*in vitro* synthesis of a novel magnetic protein. Science.

[8] Tanaka T., Matsunaga T. (2000). Fully automated chemiluminescence immunoassay of insulin using antibody-protein a-bacterial magnetic particle complexes. Anal. Chem.

[9] Tamer U., Gundogdu Y., Boyaci I.H., Pekmez K. (2010). Synthesis of magnetic core-shell Fe_3_O_4_-Au nanoparticle for biomolecule immobilization and detection. J. Nanopart. Res.

[10] Mirkin C.A., Letsinger R.L., Mucic R.C., Storhoff J.J. (1996). A DNA-based method for rationally assembling nanoparticles into macroscopic materials. Nature.

[11] Lin J., Zhou W., Kumbhar A., Wiemann J., Fang J., Carpenter E.E., O’Connor C.J. (2001). Gold-coated iron (Fe@Au) nanoparticles: Synthesis, characterization, and magnetic field-induced self-assembly. J. Solid State Chem.

[12] Cho S.-J., Idrobo J.-C., Olamit J., Liu K., Browning N.D., Kauzlarich S.M. (2005). Growth mechanism and oxidation resistance of gold-coated iron nanoparticles. Chem. Mater.

[13] Mikhaylova M., Kim D.K., Bobrysheva N., Osmolowsky M., Semenov V., Tsakalakos T., Muhammed M. (2004). Superparamagnetism of magnetite nanoparticles: Dependence on surface modification. Langmuir.

[14] Mandal M., Kundu S., Ghosh S.K., Panigrahi S., Sau T.K., Yusuf S.M., Pal T. (2005). Magnetite nanoparticles with tunable gold or silver shell. J. Colloid Interf. Sci.

[15] Wang L.Y., Luo J., Maye M.M., Fan Q., Qiang R.D., Engelhard M.H., Wang C.M., Lin Y.H., Zhong C.J. (2005). Iron oxide-gold core-shell nanoparticles and thin film assembly. J. Mater. Chem.

[16] Wang L.Y., Luo J., Fan Q., Suzuki M., Suzuki I.S., Engelhard M.H., Lin Y.H., Kim N., Wang J.Q., Zhong C.J. (2005). Monodispersed core-shell Fe_3_O_4_@Au nanoparticles. J. Phys. Chem. B.

[17] Pham T.T.H., Cao C., Sim J. (2008). Application of citrate-stabilized gold-coated ferric oxide composite nanoparticles for biological separations. J. Magn. Magn. Mater.

[18] Carpenter E.E., Kumbhar A., Wiemann J.A., Srikanth H., Wiggins J., Zhou W.L., O’Connor C.J. (2000). Synthesis and magnetic properties of gold-iron-gold nanocomposites. Mat. Sci. Eng.

[19] Carpenter E.E. (2001). Iron nanoparticles as potential magnetic carriers. J. Magn. Magn. Mater.

[20] Zhou W.L., Carpenter E.E., Lin J., Kumbhar A., Sims J., O’Connor C.J. (2001). Nanostructures of gold coated iron core-shell nanoparticles and the nanobands assembled under magnetic field. Eur. Phys. J. D.

[21] Jeong J., Ha T.H., Chung B.H. (2006). Enhanced reusability of hexa-arginine-tagged esterase immobilized on gold-coated magnetic nanoparticles. Anal. Chim. Acta.

[22] Lyon J.L., Fleming D.A., Stone M.B., Schiffer P., Williams M.E. (2004). Synthesis of Fe oxide core/au shell nanoparticles by iterative hydroxylamine seeding. Nano Lett.

[23] Heitsch A.T., Smith D.K., Patel R.N., Ress D., Korgel B.A. (2008). Multifunctional particles: Magnetic nanocrystals and gold nanorods coated with fluorescent dye-doped silica shells. J. Solid State Chem.

[24] Tamer U., Boyaci I.H., Temur E., Zengin A., Dincer I., Elerman Y. (2011). Fabrication of magnetic gold nanorod particles for immunomagnetic separation and sers application. J. Nanopart. Res.

[25] El-Boubbou K., Gruden C., Huang X. (2007). Magnetic glyco-nanoparticles: A unique tool for rapid pathogen detection, decontamination, and strain differentiation. J. Am. Chem. Soc.

[26] Cheng Y.X., Liu Y.J., Huang J.J., Li K., Xian Y.Z., Zhang W., Jin L.T. (2009). Amperometric tyrosinase biosensor based on fe3o4 nanoparticles-coated carbon nanotubes nanocomposite for rapid detection of coliforms. Electrochim. Acta.

[27] Varshney M., Li Y.B. (2007). Interdigitated array microelectrode based impedance biosensor coupled with magnetic nanoparticle-antibody conjugates for detection of escherichia coli o157 : H7 in food samples. Biosens. Bioelectron.

[28] Chen W., Shen H.B., Li X.Y., Jia N.Q., Xu J.M. (2006). Synthesis of immunomagnetic nanoparticles and their application in the separation and purification of cd34(+) hematopoietic stem cells. Appl. Surf. Sci.

[29] Gupta A.K., Curtis A.S.G. (2004). Lactoferrin and ceruloplasmin derivatized superparamagnetic iron oxide nanoparticles for targeting cell surface receptors. Biomaterials.

[30] Premasiri W.R., Moir D.T., Klempner M.S., Krieger N., Jones G., Ziegler L.D. (2005). Characterization of the surface enhanced raman scattering (SERS) of bacteria. J. Phys. Chem. B.

[31] Sengupta A., Mujacic M., Davis E.J. (2006). Detection of bacteria by surface-enhanced raman spectroscopy. Anal. Bioanal. Chem.

[32] Kahraman M., Yazici M.M., Sahin F., Culha M (2007). Experimental parameters influencing surface-enhanced Raman scattering of bacteria. J. Biomed. Opt..

[33] Marotta N.E., Bottomley L.A. (2010). Surface-enhanced Raman scattering of bacterial cell culture growth media. Appl. Spectrosc.

[34] Guven B., Basaran-Akgul N., Temur E., Tamer U., Boyaci I.H. (2011). SERS-based sandwich immunoassay using antibody coated magnetic nanoparticles for escherichia coli enumeration. Analyst.

[35] Wannapob R., Kanatharana P., Limbut W., Numnuam A., Asawatreratanakul P., Thammakhet C., Thavarungkul P. (2010). Affinity sensor using 3-aminophenylboronic acid for bacteria detection. Biosens. Bioelectron.

[36] Ciftci H., Tamer U. (2011). Electrochemical determination of iodide by poly(3-aminophenylboronic acid) film electrode at moderately low ph ranges. Anal. Chim. Acta.

[37] Ertl P., Mikkelsen S.R. (2001). Electrochemical biosensor array for the identification of microorganisms based on lectin-lipopolysaccharide recognition. Anal. Chem.

[38] Lau O.W., Shao B., Lee M.T.W. (2000). Affinity mass sensors: Determination of fructose. Anal. Chim. Acta.

[39] Yonzon C.R., Haynes C.L., Zhang X.Y., Walsh J.T., van Duyne R.P. (2004). A glucose biosensor based on surface-enhanced Raman scattering: Improved partition layer, temporal stability, reversibility, and resistance to serum protein interference. Anal. Chem.

[40] Suo Z.Y., Yang X.H., Avci R., Deliorman M., Rugheimer P., Pascual D.W., Idzerda Y. (2009). Antibody selection for immobilizing living bacteria. Anal. Chem.

[41] Premkumar J.R., Lev O., Marks R.S., Polyak B., Rosen R., Belkin S. (2001). Antibody-based immobilization of bioluminescent bacterial sensor cells. Talanta.

[42] Temur E., Zengin A., Boyaci I.H., Dudak F.C., Torul H., Tamer U. (2012). Attomole sensitivity of staphylococcal enterotoxin B detection using an aptamer-modified surface-enhanced raman scattering probe. Anal. Chem.

