# Supplementary material for: Gold-Coated Iron Composite Nanospheres Targeted the Detection of Escherichia coli
Source: Int J Mol Sci. 2013 Mar 18;14(3):6223–40. doi: 10.3390/ijms14036223 (PMC3634437; doi:10.3390/ijms14036223)
Supplement: Supplementary file 1 [file ijms-14-06223-s001.doc]

Supplementary Information

**Figure S1.** (**a**) AFM images of gold coated magnetic nanoparticles; (**b**) Particle radius histograms of this sample.


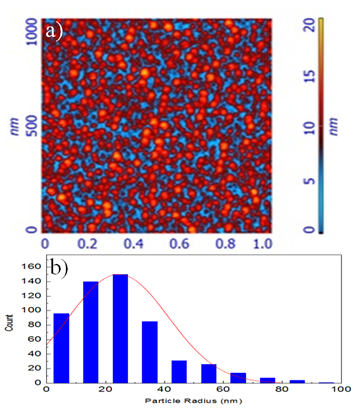


**Figure S2.** UV-Vis absorption spectra for magnetic gold nanosphere particles.


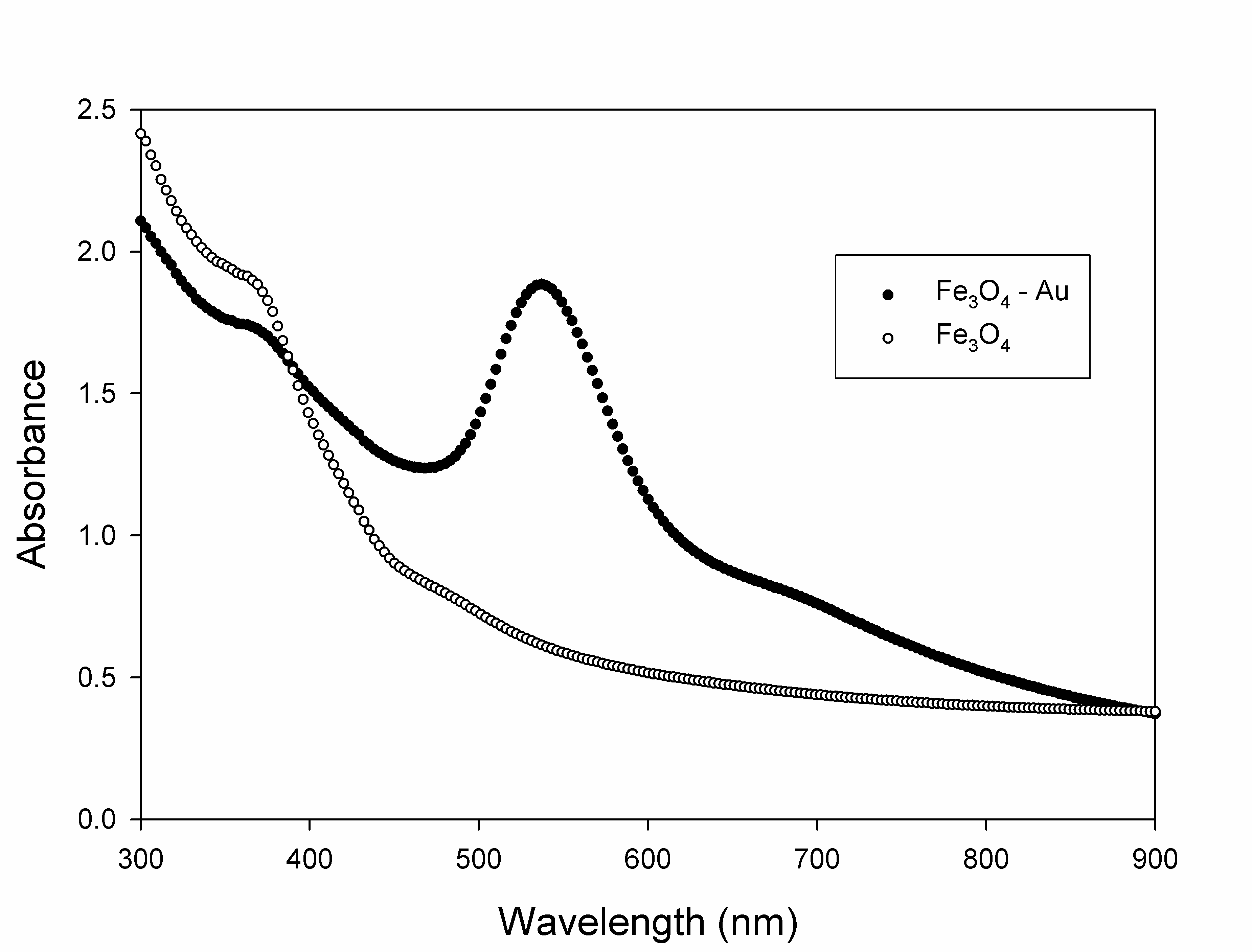


**Figure S3.** SERS spectrum of (**a**) 10^7^ cfu mL^−1^ *E. coli*, (**b**) 3-MBA/1-DT-modified magnetic nanoparticles and (**c**) *E. coli* interacted with 3-MBA/1-DT-modified magnetic nanoparticles.


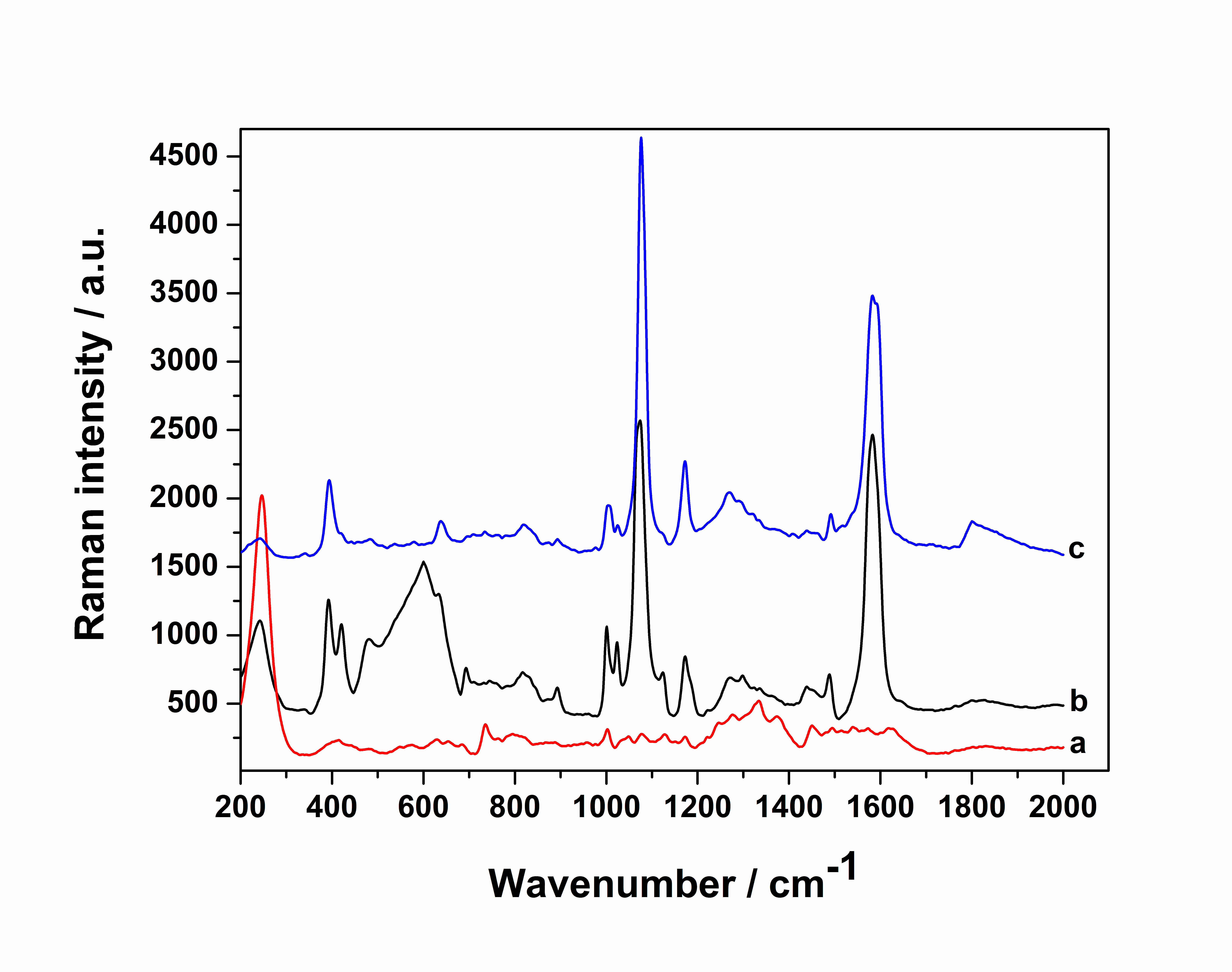


**Figure S4.** SERS spectrum of (**a**) 10^7^ cfu mL^−1^ *E. coli*, (**b**) 3-MBA modified magnetic nanoparticles and (**c**) *E. coli* interacted with 3-MBA modified magnetic nanoparticles.


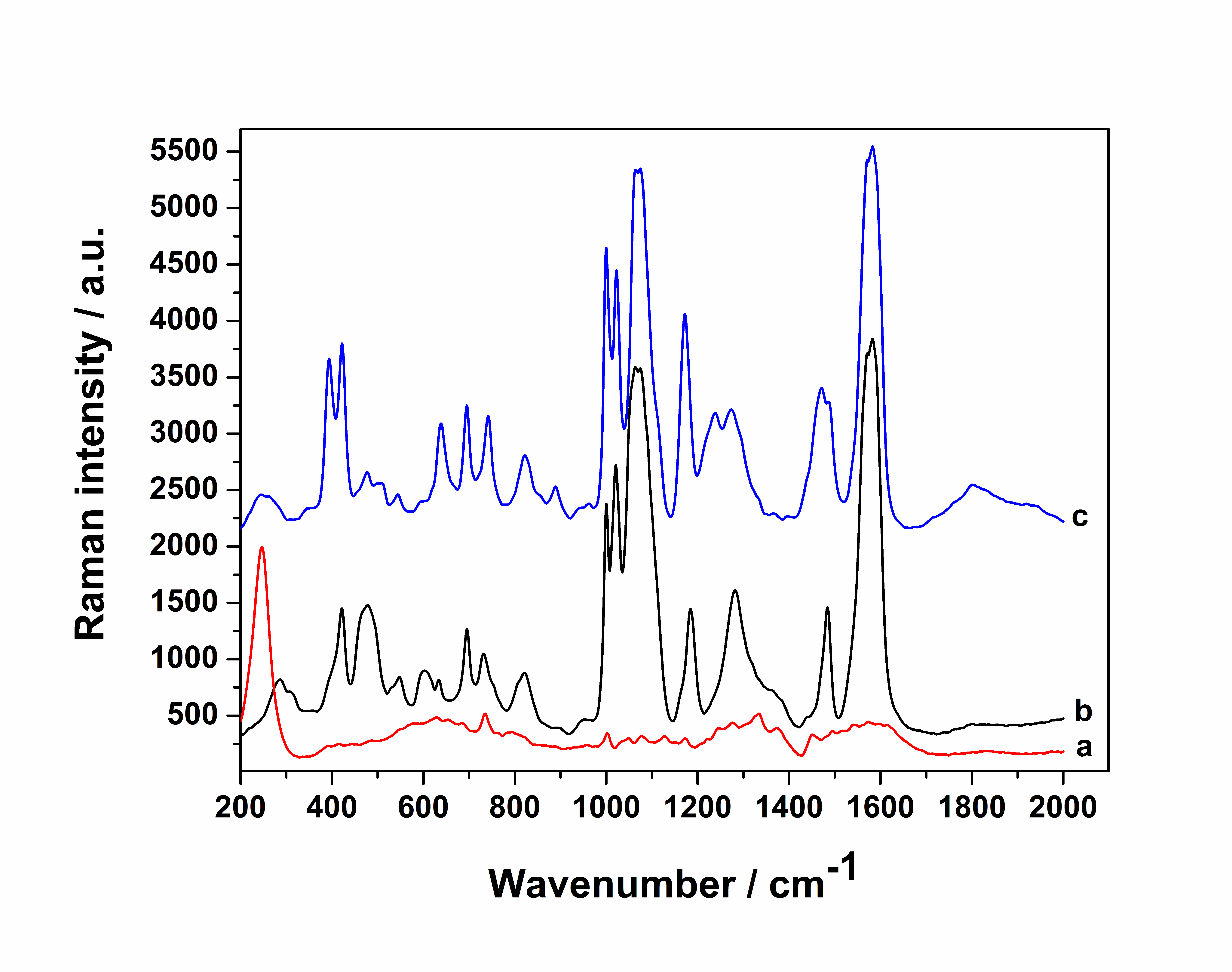


**Figure S5.** SERS spectrum of (**a**) 10^7^ cfu mL^−1^ *E. coli*, (**b**) 1-DT-modified magnetic nanoparticles and (**c**) *E. coli* interacted with 1-DT-modified magnetic nanoparticles.


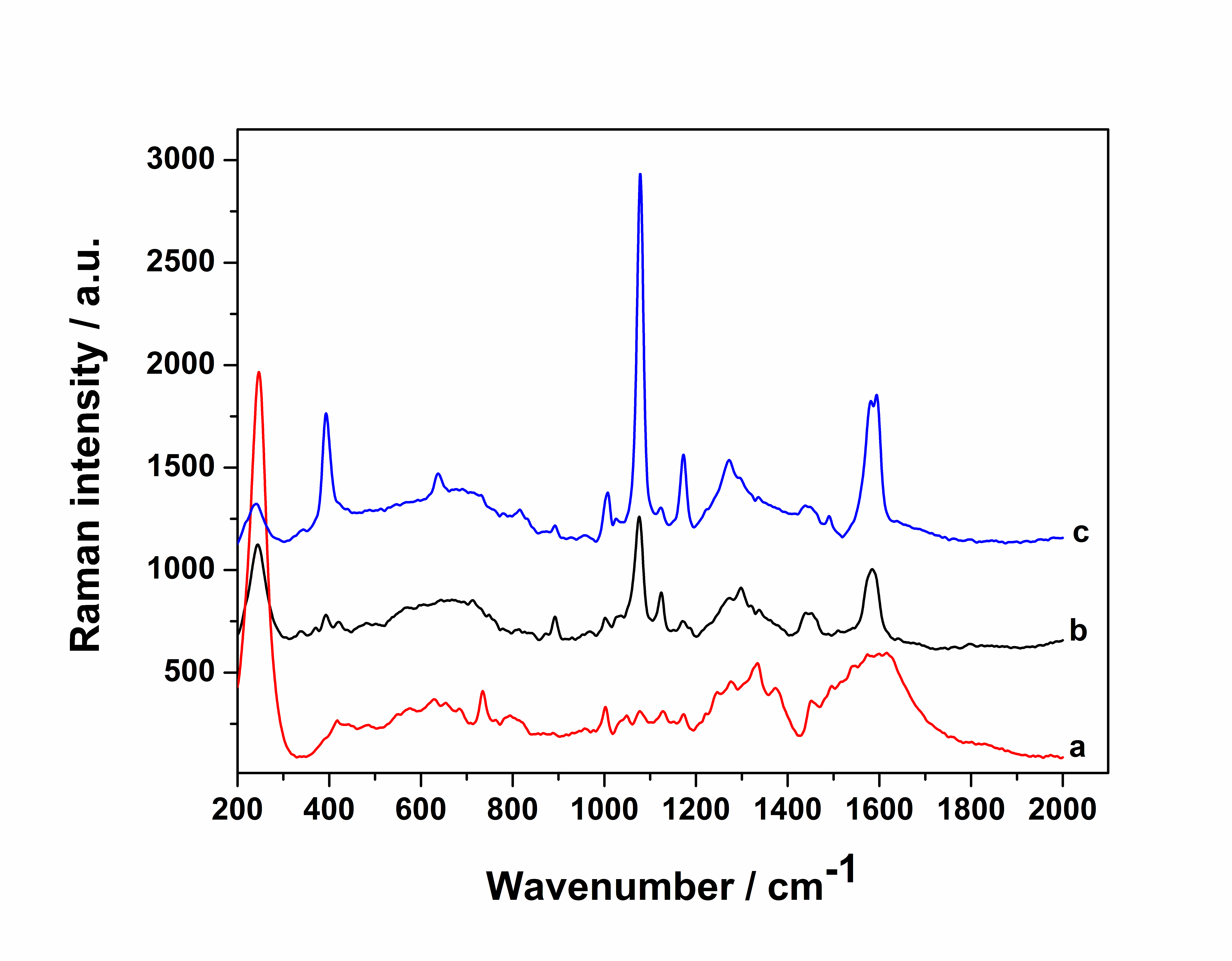


**Figure S6.** SERS spectrum of (**a**) 10^7^ cfu mL^−1^ *E. coli*, (**b**) CTAB modified magnetic nanoparticles and (**c**) *E. coli* interacted with CTAB modified magnetic nanoparticles.


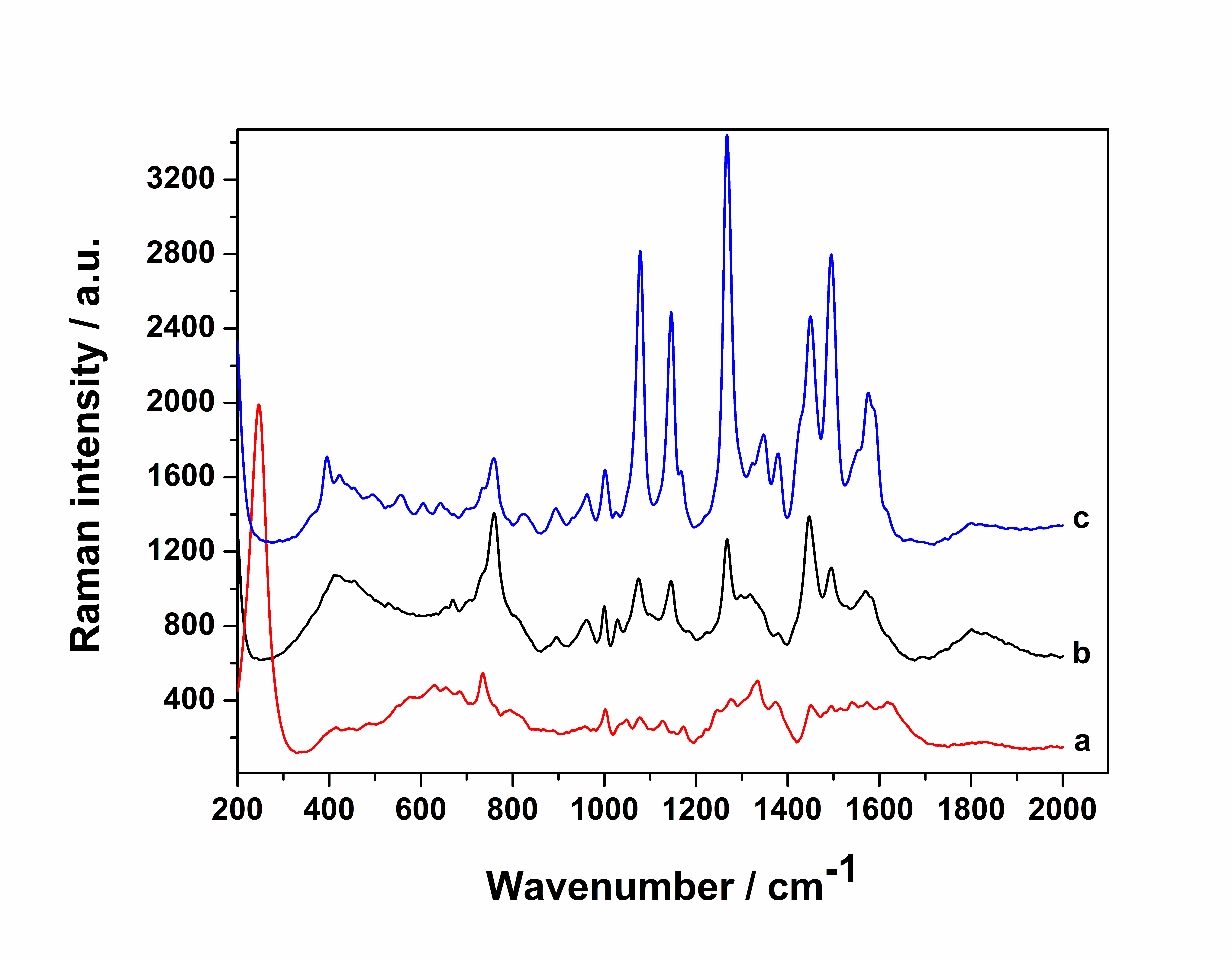


© 2013 by the authors; licensee MDPI, Basel, Switzerland. This article is an open access article distributed under the terms and conditions of the Creative Commons Attribution license (http://creativecommons.org/licenses/by/3.0/).
